# The intratumor microbiota and thyroid cancer: a review

**DOI:** 10.3389/fendo.2025.1536155

**Published:** 2025-06-30

**Authors:** Shan Liu, Xiaoliang Xiong, Tingting Hao, Xue Shi, Yinlong Zhao

**Affiliations:** Department of Nuclear Medicine, The Second Hospital of Jilin University, Changchun, China

**Keywords:** intratumor microbiota, thyroid cancer, tumorigenesis, mechanisms, tumor microenvironment

## Abstract

Thyroid cancer is a prevalent malignancy with a rising global incidence, driven by factors such as genetic mutations, environmental influences, and gender differences. Despite advancements in diagnostic techniques and treatments, effective therapies for advanced or iodine-refractory thyroid cancer remain limited. Recent discoveries have challenged the long-held belief that tumors are sterile, revealing the presence of intratumor microbiota in various cancers. Intratumor microbiota significantly impact cancer development, immune reactions, and the effectiveness of treatments. This review examines the emerging evidence of intratumor microbiota in thyroid cancer, emphasizing their potential roles in tumor development. We also examine the origins and diversity of these microbial communities and investigate the mechanisms through which they might affect thyroid cancer progression. Understanding the complex interactions between intratumor microbiota and thyroid cancer could inform the development of novel diagnostic tools and therapeutic strategies to improve patient outcomes.

## 1 Introduction

Thyroid cancer is a prevalent public health issue with a steadily increasing incidence worldwide in recent decades. According to Global Cancer Statistics 2024, an estimated 44,020 new cases and 2,170 deaths occurred in the United States (1). Over the past four decades, thyroid cancer incidence has risen by 313%, largely due to the enhanced detection capabilities afforded by the extensive use of imaging studies and the advent of fine-needle aspiration biopsies (2). Thyroid cancer is categorized into four primary histological subtypes based on cellular origin, molecular pathogenesis, clinical presentation, and progression: papillary thyroid cancer (PTC), follicular thyroid cancer (FTC), medullary thyroid cancer (MTC), and anaplastic thyroid cancer (ATC). PTC is the most prevalent type, accounting for 80-85% of all thyroid cancer cases, followed by FTC (10-15%), MTC (3-5%), and ATC (<2%) (3). Thyroid cancer development is affected by both genetic and environmental factors. Numerous risk factors for thyroid cancer have been recognized, including exposure to ionizing radiation (4), iodine imbalance, familial thyroid cancer history, and specific genetic syndromes. Additionally, gender may influence thyroid cancer susceptibility, with a notable predominance in females who are three times more likely to develop the disease compared to males (5) and involve a multifaceted interplay of genetic and epigenetic alterations. Thyroid cancer development and progression have been linked to mutations in key genes including *BRAF*, *RAS*, *RET*, and *P53* (6, 7). These mutations activate oncogenic signaling pathways such as MAPK and PI3K/AKT, facilitating cell proliferation, survival, and invasion (6, 8). Epigenetic alterations, such as DNA methylation (9) and histone modifications (10), have been implicated in thyroid carcinogenesis. Surgery remains an effective treatment for patients with a suspected or cytologically confirmed differentiated thyroid cancer (DTC) which consistently exhibits high survival rates. The administration of radioactive iodine post-surgery enhances overall survival among patients at elevated risk of recurrence. The utilization of antiangiogenic multikinase inhibitors (eg, sorafenib, lenvatinib, cabozantinib) and therapies targeted at genetic mutations responsible for thyroid cancer is on the rise in the management of metastatic disease (11). The discovery of new biomarkers for thyroid cancer has significantly advanced understanding of its molecular pathogenesis, enabling the creation of more personalized treatment approaches for affected patients. Despite advancements in molecular testing and the discovery of promising therapies, effective treatments for advanced metastatic iodine-refractory thyroid cancer are still lacking. Thus, its diverse subtypes and complex pathogenesis necessitate a comprehensive understanding to optimize management and improve patient outcomes.

Humans harbor a vast and diverse community of microorganisms including bacteria, fungi, viruses, and archaea collectively known as the human microbiota (12). These microbes reside on our skin, in our digestive system, respiratory system, and reproductive and in various other body sites, which is increasingly recognized as a key factor in health and disease (13). Recent decades have seen substantial advancements in comprehending the microbiota-cancer interactions, uncovering intricate relationships that affect cancer development, progression, and treatment outcomes (14, 15). Research on the causal link between microbiota and cancer mainly concentrates on gut microbiota (16, 17). With the improvement of genome sequencing over the past decade, intratumor microbiota have been detected within the microenvironment of various solid tumors, challenging the traditional view of tumors as sterile entities (18, 19). Recent research has illuminated the presence and potential impact of intratumor microbiota and this emerging field of study is rapidly gaining momentum, revealing the intricate interplay between these microbial inhabitants and cancer. Intratumor microbiota, integral to the tumor microenvironment (TME), significantly influence cancer initiation, progression, and therapeutic responses by modulating immune responses and metabolic pathways (20, 21). Hitherto, intratumor microbiota have been identified in a variety of cancers, such as colorectal, pancreatic, bladder gastric, breast, lung, ovarian, prostate, and thyroid cancers (22–25). However, research examining the relationship between thyroid cancer and intratumor microbiota remains limited. Herein, we review the origin and diversity of intratumor microbiota, summarize current findings on their role in thyroid cancer, and explore the mechanisms by which they may influence cancer development. This review aims to inform and inspire future research in this emerging field.

## 2 Intratumor microbiota

The investigation of intratumor microbiota has a protracted history, originating with early documentation of tumor-associated infections in antiquity (20). Bacteria were initially identified within human tumors over a century ago (26); however, the comprehensive characterization of the intratumor microbiota has been impeded by its low biomass and constrained diagnostic tools. Advances in sequencing technologies, particularly next-generation sequencing and more recently third-generation sequencing (e.g., Nanopore and PacBio) in the 21st century have facilitated a greater recognition of the presence and significance of microbiota within tumors at an unprecedented depth. This progress has enabled extensive studies that have delineated the diversity, spatial distribution, and potential roles of these microbiota in cancer diagnosis and prognosis (18, 19). In 2020, Poore et al. performed a comprehensive study on intratumor microbiota across over 30 cancer types, revealing significant associations between specific microbiota and various cancers. Their findings suggested that microbial-based cancer diagnostics may offer significant future value to patients (18). Simultaneously, another comprehensive study conducted in 2020 demonstrated that intratumor microbiota predominantly reside intracellularly, being located within both cancerous and immune cells (19). It has been observed that the microbial community within a tumor exhibits a non-random distribution; rather, it was precisely structured into microniches linked to immune and epithelial cell functions that influenced cancer progression (27). A comprehensive analysis of 17,401 samples spanning 35 cancer types has identified the presence of low-abundance fungal DNA and cells across various malignancies (28). Additionally, the findings indicated that these fungal communities coexisted with bacterial populations and immune cells within TME, potentially influencing these niches (28). Intratumor microbiota may actively contribute to tumorigenesis and cancer progression through mechanisms such as direct genotoxicity, immune response modulation, and metabolic reprogramming (20, 29). For example, certain microbiota produce genotoxins that damage host DNA, potentially leading to mutations that drive cancer development (30). Additionally, some microbiota could alter the TME to favor tumor growth (31). Research is ongoing into how intratumor microbiota influence the effectiveness of cancer treatments.

Despite the growing body of evidence supporting the presence of intratumor microbiota, this field faces substantial methodological challenges, particularly concerning the risk of contamination. The detection of microbial DNA in tumor tissues—especially those with low microbial biomass—raises important questions about the authenticity of these findings. Contamination can arise from multiple sources, including laboratory reagents, environmental exposure during sample handling, and sequencing platforms themselves. This issue is especially pronounced in studies utilizing formalin-fixed paraffin-embedded tissues, where both the degradation of nucleic acids and the introduction of exogenous microbial DNA during processing can confound results. Recent studies have underscored the critical importance of implementing robust contamination control measures (32). These include the use of appropriate negative controls (e.g., blank extractions, reagent-only controls), rigorous sterilization procedures during sample collection and processing, and the application of bioinformatic techniques to distinguish true microbial signals from background noise. Moreover, low-biomass microbiome studies demand specialized protocols to minimize and monitor contamination at every step-from DNA extraction to sequencing and data analysis (33). In conclusion, while the presence of intratumor microbiota has been supported by multiple independent studies employing diverse methodologies, continued efforts to standardize protocols and improve contamination control are essential to advance this emerging field and validate its translational potential.

### 2.1 The potential origins of intratumor microbiota

**Figure 1:**
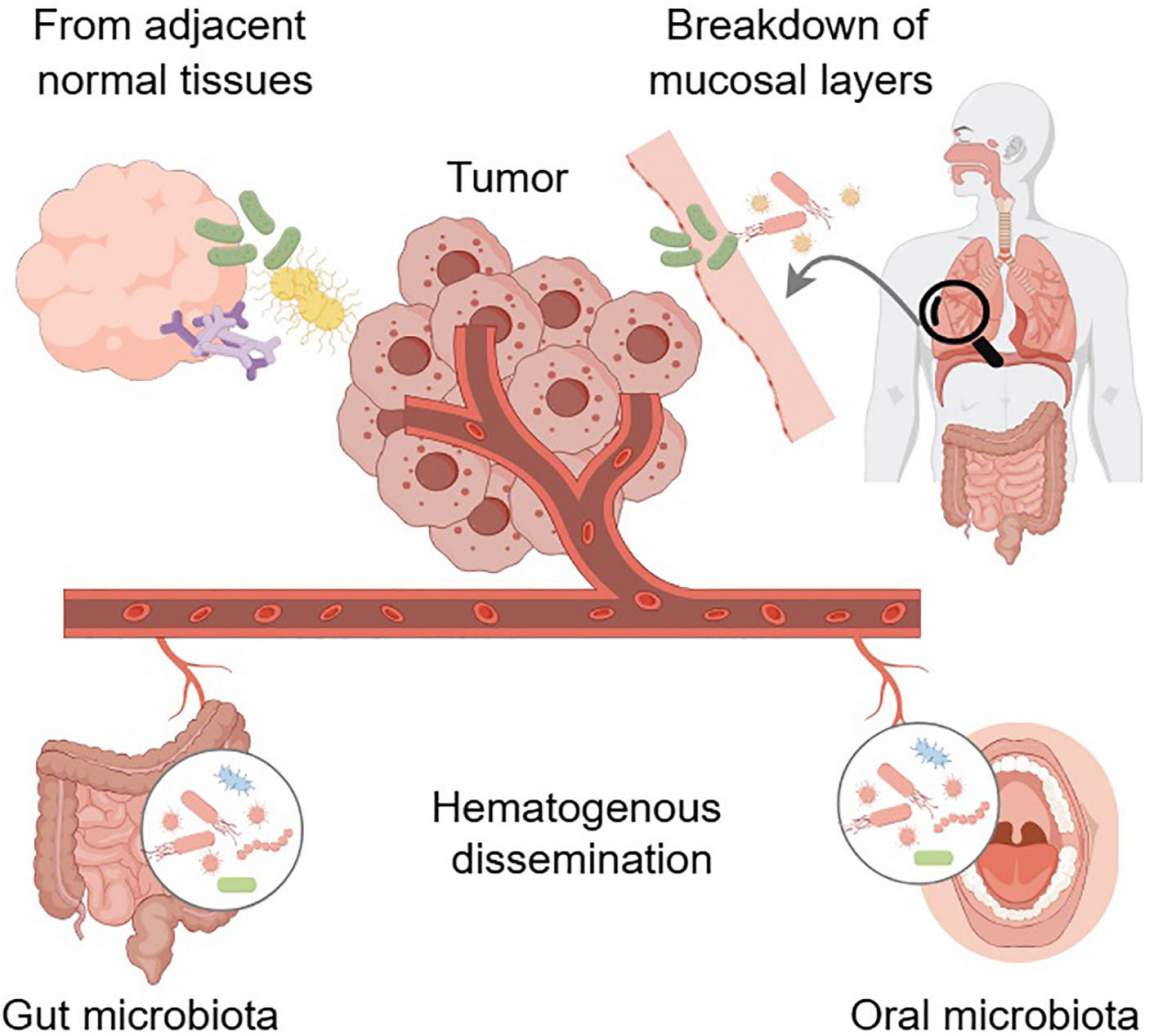
The potential origins of intratumor microbiota. Hematogenous dissemination may facilitate the infiltration of microbes from distant sites, such as the oral cavity and gut, into the tumor. Translocation of bacteria from adjacent normal tissues represents another potential origin. Disruption of mucosal barriers in organ cavities exposed to the external environment could allow resident microbes to penetrate the tumor. Arrows indicate the direction of the effect. Arrows with dashed lines indicate that no studies have explored this putative relationship yet. Graphics created with figdraw.com.

The origins of intratumor microbiota have not been fully elucidated and remain the focus of continued research and debate. Various hypotheses have been suggested to elucidate the mechanisms by which these microbes infiltrate and persist in the TME (**Figure 1**). Hematogenous dissemination is a potential pathway where microorganisms from distant body sites, like the mouth and intestines, enter the bloodstream and colonize tumors via damaged blood vessels (20). While direct evidence linking specific bleeding events to tumor colonization by particular microbes in humans is difficult to acquire, literature suggests that hematogenous dissemination is a plausible pathway for intratumor microbiota. Zheng et al. identified *Bacteroides* species within the tumor, as well as in the oral and intestinal microbiota, and confirmed that microbiota might migrate from the oral cavity to the intestine and ultimately to distant mammary tumor tissue (34). *Fusobacterium nucleatum*, a bacterium associated with invasive cancer, is hypothesized to migrate from the oral cavity to other body sites through the bloodstream (35, 36). Research indicated that *Escherichia coli* might contribute to colorectal cancer (CRC) metastasis to the liver to the liver by disrupting the gut vascular barrier, facilitating its entry into the bloodstream, and aiding in the establishment of a pre-metastatic niche in the liver (37). In a murine model of spontaneous breast tumors, bacteria were found within circulating tumor cells and were enriched at lung metastasis sites, suggesting that certain intracellular bacteria may spread to metastatic locations within tumor cells through the systemic circulation (38). Another potential origin is the translocation of bacteria from adjacent normal tissues. This phenomenon can be explained by the presence of low-abundance microbial communities in many tissues traditionally considered sterile. Additionally, bacteria in tumor tissues closely resemble those in nearby normal tissues (19). Bacteria from nearby normal tissues may accumulate at tumor sites during tumorigenesis due to microenvironmental changes and increased tissue accessibility caused by the disruption of epithelial and mucus barriers (36). The source of microorganisms in normal tissues is uncertain, and they might also spread from the tumor site. Therefore, this hypothesis requires further empirical validation. Intratumor microbiota are commonly found in cancers that develop in organs with mucosal surfaces, such as the colon, pancreas, cervix, and lungs. These organs feature cavities that are exposed to the external environment, and the process of tumorigenesis can disrupt the mucosal barriers, creating an opportunity for microbes residing on the mucosal surfaces to penetrate the tumor. Consequently, the breakdown of these protective mucosal layers, in conjunction with other factors, may facilitate the establishment of microbiota within the tumor. This theory suggests that the origin of intratumor microbiota in certain cancers could be attributed to the translocation of microbes from the mucosal surfaces following the loss of barrier integrity during tumor development.

Current research on the bacterial origins of thyroid cancer is limited, with studies indicating only a partial overlap in sequences between thyroid and gut microbiota (39). This limited overlap doesn’t provide sufficient evidence to suggest a link between gut and thyroid microbiota. It remains to be explored whether bacteria can migrate from the gut to the thyroid.

Understanding the precise mechanisms of microbial colonization within tumors is crucial, as these intratumor communities may significantly influence cancer progression, treatment response, and overall patient outcomes. It has been hypothesized that the TME, characterized by high hypoxia, nutrient richness, vascular hyperplasia, aerobic glycolysis, and immunosuppression, may be conducive to bacterial growth and proliferation (40). However, the specific mechanisms by which the tumor milieu serves as a suitable environment for bacterial survival and function remain unclear. Future research should explore the mechanisms that attract microorganisms to the tumor microenvironment, enable them to evade the immune system, and facilitate tumor colonization.

### 2.2 Diversity of intratumor microbiota

The diversity of intratumor microbiota may be a significant factor influencing cancer progression, treatment response, and patient prognosis. In CRC, stomach adenocarcinoma, and endometrial carcinoma, intratumor microbiota diversity correlates with microsatellite instability, which is connected to increased tumor immunity and mutational burden. Specific genera such as *Dialister* and *Castellaniella* have been correlated with improved survival rates in CRC patients, suggesting that microbiota diversity may influence both tumor immunity and mutational burden (41). Research indicates significant variation in intratumor microbiota among different cancer types. One study identified that various tumor types exhibit unique microbial compositions, and the metabolic functions encoded by these intratumor microbiota are correlated with specific clinical characteristics of certain tumor subtypes (19). Another study delineated distinct fungal communities associated with specific cancer types (28). An analysis of 32 cancer types identified unique microbial signatures linked to survival outcomes, genomic features, and immune profiles, highlighting a complex microbiota landscape within tumors (42). Guo et al. conducted a microbial analysis of three pancreatic cancer subtypes: classic, basal-like, and mixed. Their findings revealed that the basal-like subtype harbored a distinct microbial community, distinguishable from the other two subtypes through Principal Coordinates Analysis. Furthermore, the basal-like subtype exhibited a significant abundance of *Acinetobacter*, *Pseudomonas*, and *Sphingopyxis*, suggesting a potential role of these microorganisms in tumor progression (43). Liu et al. identified substantial variability in the abundance of certain CRC-associated pathogens, such as *Clostridium difficile*, *Clostridium* sp*ecies*, and *Prevotella*, across individual tumors. Additionally, they noted that the abundance of certain microorganisms within tumors can vary throughout the adenoma-carcinoma sequence (44). Despite the accumulating evidence of intratumor microbiotal diversity and its potential implications in cancer biology, current research faces several limitations. Most studies have focused on a limited number of cancer types, leaving the full extent of bacterial diversity across all cancers largely unexplored. Furthermore, the majority of these studies have utilized 16S rRNA sequencing, which offers limited resolution at the species and strain levels.

## 3 The characterization of intratumor microbiota in thyroid cancer

The “thyrogastric syndrome” concept, introduced in the 1950s, is based on the embryological and physiological links between the thyroid gland and the gastrointestinal tract (45). The thyroid gland originates from the endoderm, specifically from the floor of the primitive pharynx, which constitutes a part of the foregut. Thyroid follicular cells and gastric mucosal cells originate from the same endodermal embryonic layer (46). This shared developmental origin has prompted researchers to investigate potential similarities and interactions, including the possibility of microbial colonization within the thyroid gland. Based on this connection, it is reasonable to hypothesize the thyroid gland may harbor microorganisms essential for various physiological functions. Although research on intratumor microbiota in thyroid cancer is less extensive than in other cancers, this emerging field is showing promising progress (**Table 1**). Recent studies reveal distinct microbial compositions in thyroid cancer tumors compared to nearby peritumor tissues, suggesting a potential role of microbiota in the development and progression of thyroid cancer. In a groundbreaking study, Dai et al. examined microbiota changes in different thyroid microhabitats in thyroid cancer patients (47). The study identified significant variations in microbiota composition and diversity between tumor and peritumor tissues. Specifically, the core microbiota of the thyroid comprised *Sphingomonas*, *Comamonas*, *Acinetobacter*, *Pseudomonas*, *Microvirgula*, and *Soonwooa*. The study demonstrated a notable rise in *Sphingomonas* and *Aeromonas* in tumor tissues, whereas *Comamonas*, *Acinetobacter*, and *Peptostreptococcus* were more common in peritumor tissues (47). In a recent study, 109 microbial species were found to be significantly altered when comparing tumor and adjacent normal tissues in PTC. Among these, 14 fungal species were predominantly found in tumor tissues, while 94 fungal species and one archaeal species were more prevalent in normal tissues. Fungal species such as *Metarhizium acridum CQMa 102*, *Saccharomyces cerevisiae YJM1338*, and *Phaffia rhodozyma* were notably more abundant in tumor tissues. The archaeal species *Anomalluma dodsoniana* was found to be more abundant in tumor tissue than in normal tissue. Interestingly, *Candida albicans*, *Microallomyces dendroideus*, and the archaeal species *Anomalluma dodsoniana* were predominantly observed in normal tissues (25). *Proteobacteria* were identified as the most abundant phylum among thyroid malignant tumor patients in another study (48). PTC encompasses a spectrum of histologic subtypes, including classical PTC (CPTC), follicular variant PTC (FVPTC), and tall cell PTC (TCPTC). The mycobiome analysis of PTC subtypes identified 63 fungal species with increased abundance relative to normal thyroid tissue, with FVPTC exhibiting the most significant microbial dysregulation, followed by TCPTC and CPTC (25). Among the identified fungal species, *Botrytis cinerea*, *Pichia cephalocereana*, and *Trematosphaeria pertusa* were consistently enriched in CPTC, FVPTC, and TCPTC (25). Differential archaeal abundance was observed across PTC subtypes. TCPTC exhibited enrichment of the *uncultured euryarchaeote Alv-FOS5* relative to normal tissue. FVPTC demonstrated overabundance of *uncultured marine archaeon* and *uncultured Pyrobaculum* sp. compared to normal samples. In contrast, *Halovivax ruber XH-70* and *Methanosarcina* sp. *WH1* showed reduced abundance in CPTC tumors compared to normal tissue (25). A group of microbial species, such as *Micrococcus luteus*, *Frankia* sp., *Anabaena* sp. *K119*, and *uncultured Gammaproteobacteria* were observed to be overabundant in normal tissues of these PTC subtypes (49). Notably, *Trueperella pyogenes* and *Stenotrophomonas maltophilia K279a* displayed a pattern of dysregulation that was consistent between CPTC and FVPTC (49). Each subtype revealed a distinct microbial signature: *Rhodococcus fascians-D188* was prevalent in normal CPTC samples, *Acinetobacter baumannii AB0057* in normal FVPTC samples, and *Bradyrhizobium* sp. *BTAi1* in normal TCPTC samples, underscoring the subtleties in microbial ecology that distinguish these cancer variants (49). Yuan et al. found *Pseudomonas* was the dominant bacterium in PTC, followed by *Rhodococcus*, *Ralstonia*, *Acinetobacter*, and *Sphingomonas.* The study examined microbiota alterations in PTC tumors across different stages, identifying stage-dependent variations in the abundances of the genera *Pseudomonas*, *Rhodococcus*, and *Sphingomonas*. *Pseudomonas* spp., the predominant genus across all groups, showed higher abundance in early-stage tumors (T1 and T2) than in advanced stages (T3 and T4). They also found *Rhodococcus* was significantly more abundant in patients with T1 PTC compared to those with T3 PTC, while *Sphingomonas* showed higher abundance in T1 and T2 than in T3. The T1_2 tumors predominantly featured the genera *Pseudomonas*, *Rhodococcus*, and *Sphingomonas*.T3_4 tumors predominantly featured the genera *Streptococcus*, *Granulicatella*, *Haemophilus*, and *unclassified Rhizobiales*, along with *unranked Coriobacteriales* (48). Notably, the number of microorganisms diminished as the distance from the cancerous tissue increased (39). Sex-specific differences in intratumor microbiota were also observed. *Synechococcus* sp. *CC9311* was found to be overabundant in normal samples among males, whereas it was overabundant in tumor samples among females (49). The term alpha diversity(α-diversity) characterizes the richness and evenness of microbial populations in a specific ecological environment (50). The α-diversity of the intratumor microbiota was significantly lower in males compared to females, although no significant differences in β-diversity were found between sexes (48). The genera *Rhodococcus*, *Ralstonia*, *Chryseobacterium*, and *Burkholderia-Caballeronia*, *Paraburkholderia* were observed to be more common in females compared to males (48). A separate study found a significant alteration in the abundance of 88 fungal species in females, whereas only 11 fungal and archaeal species showed differential abundance in males (25). These findings collectively underscored the intricate and dynamic characteristics of intratumor microbiota in thyroid cancer. Distinct microbial profiles associated with different PTC subtypes and stages, along with sex-specific differences, suggest that intratumor microbiota could be crucial in thyroid cancer development and progression. Additional studies are required to elucidate the functions of these microbial communities and assess their potential as diagnostic biomarkers or therapeutic targets.

**Table 1:** The characterization of intratumor microbiota in thyroid cancer.

Year of study	Number of clinical samples	Methods	Main findings	Reference
2021	Tumor tissues and matched peritumor tissues from 30 patients with thyroid cancer	16s rRNA gene sequencing	The core microbiota of thyroid included *Sphingomonas*, *Comamonas*, *Acinetobacter*, *Pseudomonas*, *Microvirgula*, and *Soonwooa*. *Sphingomonas* and *Aeromonas* were significantly enriched in tumor tissues, whereas *Comamonas*, *Acinetobacter*, and *Peptostreptococcus* were markedly increased in peritumoral tissues	(47)
2021	563 thyroid cancer patients (354 CPTC, 101 FVPTC, 35 TCPTC, 135 male, 366 female tumor samples	TCGA	*Micrococcus luteus*, *Frankia* sp., *Anabaena* sp. *K119*, and an *uncultured Gammaproteobacterium* were all found to be similarly enriched in the normal tissues of CPTC, FVPTC, and TCPTC. *Trueperella pyogenes* and *Stenotrophomonas maltophilia K279a* exhibited comparable dysregulation in both CPTC and FVPTC. *Rhodococcus fascians* D188 showed increased abundance in the normal samples from CPTC, while *Acinetobacter baumannii AB0057* was more abundant in the normal samples from FVPTC. *Bradyrhizobium* sp. *BTAi1* was enriched in the normal tissues of TCPTC. For *Synechococcus* sp. *CC9311*, higher abundance was observed in normal samples from males, whereas in females, it was more prevalent in tumor samples	(49)
2021	the 93 samples from thyroid patients (19 malignant and six benign patients)	16s rRNA gene sequencing	*Proteobacteria* constitutes the most abundant bacterial phylum in thyroid cancer tissue, while *Actinobacteria* is the most abundant phylum in para-tumor tissue	(39)
2022	Tumor samples from 80 patients with PTC	16s rRNA gene sequencing	*Pseudomonas* was the dominant bacterium, followed by *Rhodococcus*, *Ralstonia*, *Acinetobacter*, and *Sphingomonas*. Rhodococcus, Ralstonia, Chryseobacterium, and Burkholderia-Caballeronia, Paraburkholderia were found to be more prevalent in females than in males. *Pseudomonas* spp., the most abundant genus in all groups, were more abundant in early-stage tumors (T1 and T2) compared to advanced stages (T3 and T4). *Rhodococcus* abundance was also significantly higher in patients with T1 PTC than in those with T3 PTC, and *Sphingomonas* was more abundant in T1 and T2 than in T3. The T1_2 tumors exhibited a predominance of *Pseudomonas*, *Rhodococcus*, and *Sphingomonas*. T3_4 tumors were dominated by *Streptococcus*, *Granulicatella*, *Haemophilus g_unclassified_o_Rhizobiales*, and *g_norank_f_norank_o_-Coriobacteriales.*	(48)
2023	453 primary tumor tissue samples and 54 adjacent solid tissue normal samples	TCGA	The fungal species Metarhizium acridum CQMa 102, *Saccharomyces cerevisiae YJM1338*, and *Phaffia rhodozyma* were found to be overabundant in PTC tumor tissue, as opposed to adjacent normal tissue. The archaeal species *Anomalluma dodsoniana* was overrepresented in PTC tumor tissue compared to normal. A greater number of species were abundant in normal tissue, including *Candida albicans* and *Microallomyces dendroideu*s, along with the archaeal species *Anomalluma dodsoniana*. A total of 88 fungal microbes exhibited significant dysregulation exclusively in females, while only 11 fungal and archaeal microbes showed significant dysregulation exclusively in males	(25)

*CPTC,* classical papillary thyroid cancer; *FVPTC,* follicular variant papillary thyroid cancer; *PTC,* papillary thyroid cancer; *TCGA,* The Cancer Genome Atlas; *TCPTC,* tall cell papillary thyroid cancer.

## 4 The role of intratumor microbiota in thyroid cancer

Growing evidence indicates that the intratumor microbiota may influence thyroid cancer development and progression. Recent research suggested that certain bacterial species present in tumors could act as biomarkers for diagnosing and predicting thyroid cancer outcome. A study conducted by Dai et al. served as an example. The combination of *Comamonas* and *Sphingomonas* had been identified as an effective biomarker for differentiating between tumor and peritumor tissues. A greater presence of *Sphingomonas* was associated with lymph node metastasis, indicating its potential as a prognostic marker in thyroid cancer patients. These findings strongly suggested that *Sphingomonas* may be actively involved in promoting thyroid cancer progression (47). In addition to serving as potential biomarkers, the intratumor microbiota has also been linked to various clinical parameters that are commonly used to assess tumor aggressiveness, surgical outcomes, and risk stratification in patients with DTC. These parameters, collectively known as the distant metastasis, patient age, completeness of excision, invasion, and tumor size (MACIS) classification, provide valuable information for guiding treatment decisions and predicting patient outcomes (51). Notably, certain intratumor microbiota species has demonstrated significant correlations with MACIS scores and pathologic M stage in patients with PTC. Specifically, *Frankia* sp. and *uncultured Gammaproteobacteria* bacterium, which predominated in all PTC normal tissue samples, was linked to lower MACIS scores, indicating a possible protective function. In contrast, *Bradyrhizobium* sp. *BTAi1*, uniquely in TCPTC normal tissue, had been correlated with higher MACIS scores, indicating an association with more aggressive disease. Moreover, *Fran kia* sp. and *Anabaena* sp. *K119*, overabundant in normal tissue samples of all PTC subtypes, negatively correlates with pathologic M stage, suggesting a protective role against metastasis. Conversely, *Stenotrophomonas maltophilia*, found to be dysregulated exclusively in CPTC and FVPTC, is positively associated with pathologic M stage, suggesting a potential connection to enhanced metastatic potential (49). John et al. identified correlations between specific fungal abundances and pathological staging in PTC. The abundance of *Chaetomium globosum CBS 148.51* was positively correlated with advancing pathological stage. Furthermore, they found 18 fungal species, including *Candida albicans*, *Eremascus albus*, and *Thanatephorus cucumeris*, to be associated with an elevated pathological M stage. *Wickerhamiella pararugosa*, *uncultured Cryptomycota*, and *Spiromyces aspiralis* were also linked to a higher pathological N stage (25). The interaction between the thyroid microbiota and hormonal regulation appeared to be another critical factor in thyroid carcinogenesis. Elevated levels hormones, particularly thyroid-stimulating hormone (TSH) and thyroid hormones, had been implicated in the development of thyroid cancer (52). Specifically, in conditions such as primary hypothyroidism, compensatory increases in TSH levels can lead to thyroid hyperplasia, potentially elevating the risk of malignancy (52, 53). An intriguing discovery demonstrated that *Neisseria perflava* engages in closely related interactions with species of *Roseburia, Amaricoccus*, and *Streptomyces*, potentially coordinating a series of sequential biochemical processes involving TSH and triiodothyronine (39). In conclusion, the microbiota in thyroid cancer demonstrates multifaceted roles, serving not only as promising biomarkers but also potentially influencing disease progression and metastasis. The significant correlations between specific bacterial species and clinical parameters, including MACIS scores and metastatic stages, suggest that the microbiota plays a crucial role in influencing tumor behavior and patient outcomes. Furthermore, the complex interplay between the microbiota and thyroid hormones adds another dimension to our understanding of thyroid cancer pathogenesis. Future investigations into the functional roles of these microorganisms could potentially revolutionize therapeutic approaches and enhance personalized treatment strategies for thyroid cancer patients. The elucidation of precise mechanisms through which intratumor microbiota influence thyroid carcinogenesis remains essential for integrating microbiota-based diagnostics and interventions into clinical practice.

## 5 The potential mechanisms of intratumor microbiota affecting thyroid tumorigenesis

**Figure 2:**
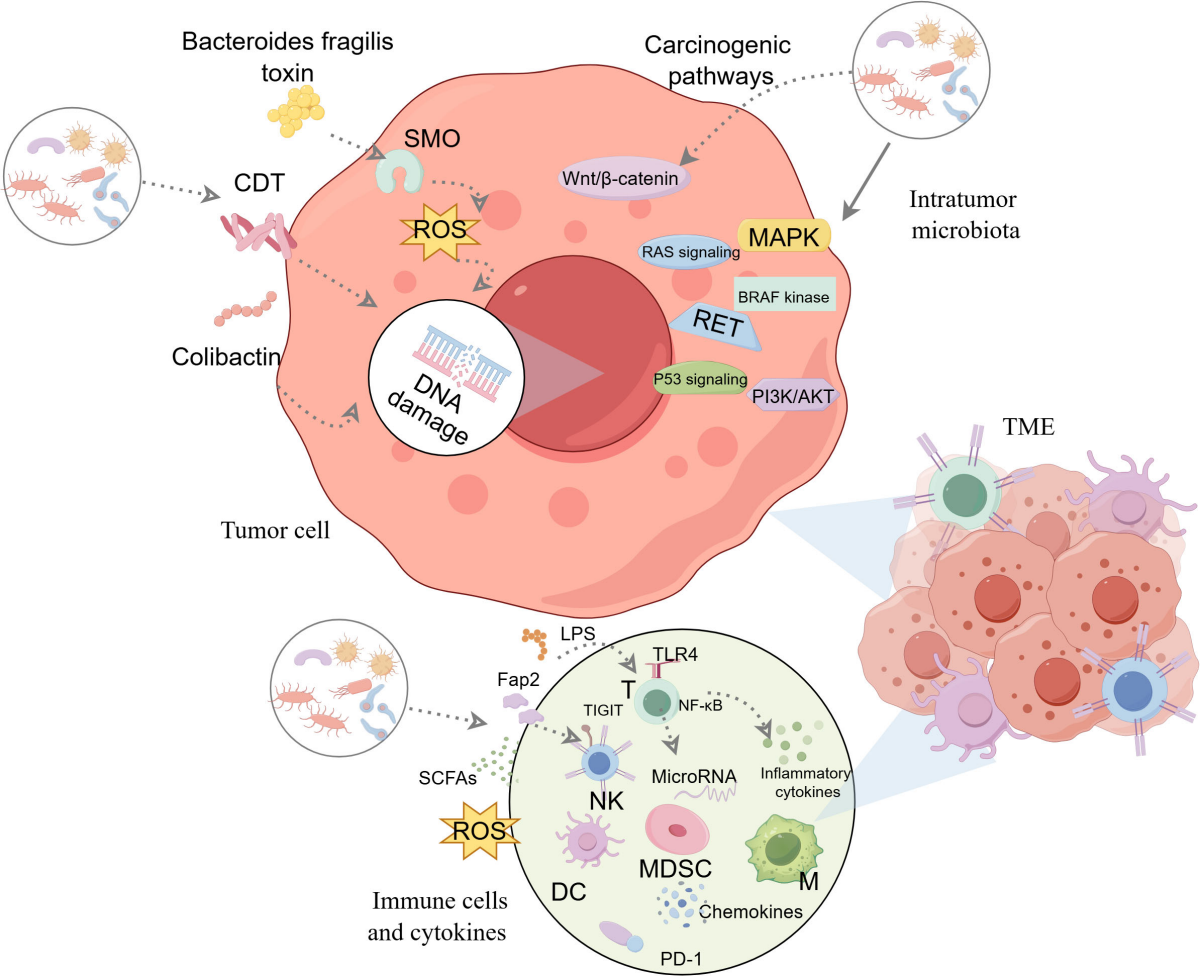
The potential mechanisms of intratumor microbiota affecting thyroid tumorigenesis. Three major potential mechanisms are thought to be involved: ①producing genotoxic substances that directly cause DNA damage and promote carcinogenesis.②regulating oncogenic signaling pathways.③altering the tumor immune microenvironment and modulating the immune response. Graphics created with figdraw.com. CDT, cytolethal distending toxin; DC, dendritic cells; LPS, lipopolysaccharide; M, macrophages; MDSC, myeloid-derived suppressor cells; NF-κB, nuclear factor-kappaB; NK, natural killer; ROS, reactive oxygen species; SCFAs, short-chain fatty acids; SMO, spermine oxidase; T, T-lymphocyte; TIGIT, T cell immunoglobulin and ITIM domain; TLRs, toll-like receptors; TME, tumor microenvironment; PD-1, Programmed death-1.

While the presence and diversity of intratumor microbiota in thyroid cancer are becoming increasingly recognized (47, 49), the precise mechanisms through which they influence tumor development and progression remain largely unexplored. The role of intratumor microbiota in thyroid cancer remains under-researched compared to other cancer types, highlighting a significant gap in understanding. Three primary mechanisms are hypothesized to influence the impact of intratumor microbiota on thyroid cancer (**Figure 2**).

### 5.1 Induce DNA damage

DNA damage is a critical factor in the development of various cancers, including thyroid cancer (54, 55). Studies have showed that certain bacterial species could induce DNA damage both directly and indirectly, leading to genetic alterations that can drive carcinogenesis (56, 57). Certain bacteria, including *Escherichia coli* and *Helicobacter* pylori, produce genotoxic substances that damage DNA and promote carcinogenesis (58, 59). For instance, the bacterial genotoxin colibactin, produced by certain strains of *Escherichia coli*, could induce DNA double-strand breaks and chromosomal instability, both of which are critical events in early carcinogenesis (58, 60). Cytolethal distending toxin, a protein complex from certain gram-negative bacteria, induces DNA damage, with the CdtB subunit particularly effective at causing dose-dependent DNA breaks (21). During the breakdown of host proteins, bacteria produced harmful metabolites, such as sulfides and nitrosamines, which had been associated with causing DNA damage (61). Bacteria can also induce the production of reactive oxygen species (ROS) and reactive nitrogen species either through their own metabolism or by stimulating host immune responses. These reactive molecules may cause oxidative stress, which leads to various forms of DNA damage, including single-strand breaks, double-strand breaks, and base modifications (62, 63). Bacteroides fragilis toxin, secreted by *Bacteroides fragilis*, has been shown to enhance colon tumorigenesis by upregulating spermine oxidase (SMO), an enzyme crucial for polyamine catabolism. SMO activation increases ROS production, which in turn leads to DNA damage in colonic epithelial cells (64, 65). Some bacteria may alter host DNA repair mechanisms, resulting in greater genomic instability and an elevated risk of cancer development (66, 67). Bacteria can influence the base excision repair pathway, crucial for fixing oxidative DNA damage, thus raising the probability of mutation accumulation (66). Intratumor microbiota may facilitate thyroid cancer initiation and progression by inducing DNA damage and hindering DNA repair, leading to mutation accumulation in these pathways. Future research should investigate if genotoxin-producing bacteria contribute to DNA mutations associated with thyroid cancer.

### 5.2 Influence carcinogenic pathways

In addition to inducing DNA damage, intratumor microbiota may modulate key carcinogenic signaling pathways that affect thyroid cancer development. A study by Daniel et al. investigated the association between intratumor fungi and oncogenic pathway activity in various PTC subtypes (25). In CPTC, *Metschnikowia santaceciliae*, *Pacynthium nigrum*, *Thanatephorus cucumeris*, and *Spriromyces aspiralis* were related to downregulation of PI3K/AKT pathways. *Metschnikowia santaceciliae* and *Placynthium nigrumwere* were associated with decreased RAS signaling. In contrast, *Uncultured Galactomyces* was associated with upregulation of BRAF kinase activity, suggesting a potential oncogenic role. The study also examined TCPTC, finding that *Brevicellicium exile*, *Eremascus albus*, and *Zoophthora occidentalis* were linked to increased p53 signaling. *Metschnikowia santaceciliae* and *uncultured Glomus* were correlated with increased BRAF kinase activity. Furthermore, these two fungi, along with *Brevicellicium exile*, were associated with increased activity in the RET, MAPK, and RAS signaling pathways. In FVPTC, *uncultured Glomus* was linked to increased BRAF kinase activity and MAPK signaling, whereas *Rozella allomycis* was associated solely with increased BRAF kinase activity. The composition of the intratumor microbiota appears to be influenced by *BRAFV600E* mutation status. A surprising finding was the overrepresentation of dysregulated microbes within *BRAFV600E*-negative tumor tissue, suggesting a potential interplay between microbial communities and this specific oncogenic driver (25).

One of the most notable pathways modulated by bacteria in cancer is the Wnt/β-catenin pathway, which plays a pivotal role in regulating cell proliferation, differentiation, and apoptosis. As thyroid cancer progresses and differentiates into more aggressive forms, such as poorly differentiated thyroid cancer and ATC, additional mutations arise in key regulatory pathways, including Wnt/β-catenin, further promoting tumor growth and resistance to apoptosis (68). Rubinstein et al. showed that *Fusobacterium nucleatum* promoted colorectal carcinogenesis by modulating E-cadherin/β-catenin through via its FadA adhesin. This adhesin binds to E-cadherin on the surface of host cells, disrupting cell adhesion and leading to the activation of β-catenin, which translocated to the nucleus and activates transcription of target genes involved in cell proliferation and cancer progression (69). Further research by Rubinstein et al. showed that *Fusobacterium nucleatum* also induced the expression of Annexin A1, a Wnt/β-catenin modulator, further enhancing β-catenin signaling and promoting cancer progression (70). Enteric bacterial proteins could induce tumorigenesis by activating the β-catenin signaling pathway. Specifically, the bacterial protein AvrA was shown to enhance β-catenin signaling in colonic epithelial cells, leading to increased tumorigenesis (71). *Helicobacter pylori*, a carcinogenic bacterium linked to gastric cancer, exemplifies bacterial-induced activation of the Wnt/β-catenin pathway. Studies have shown that *Helicobacter pylorican* directly activated the Wnt/β-catenin pathway, promoting gastric carcinogenesis (72). Parida et al. demonstrated that a pro-carcinogenic colon microbe promotes breast tumorigenesis and metastasis by activating both the Notch and Wnt/β-catenin signaling axes (73). This finding indicated that bacterial activation of β-catenin signaling is not restricted to particular cancer types and may have widespread implications across various cancers, including thyroid cancer.

### 5.3 Alter the TME

The TME of thyroid cancer of various non-cancerous cells such as immune cells, fibroblasts, endothelial cells, and an extracellular matrix rich in cytokines and growth factors (74, 75). These components interact with tumor cells and influence their behavior, creating a complex, dynamic environment that promotes tumor progression (76). Intratumor microbiota actively interacts with the TME, influencing cancer progression, immune responses, and treatment efficacy. However, research on the role of intratumor microbiota in modulating the TME in thyroid cancer remains limited. Recent studies have shown that intratumor microbiota can influence the TME by modulating both innate and adaptive immune responses (31).One study identified that CPTC exhibited the highest absolute number of correlations between intratumor microbiota dysbiosis and the dysregulation of immune-related genes. Additionally, it suggested that the intratumor microbiota might influence CD4+ T cells and helper T cells to mount a defense against tumor progression in FVPTC (49). In a study of melanoma tissues, a negative correlation was observed between the abundance of certain bacterial genera, including *Algibacter* and *Epilithonimonas*, and the infiltration of CD8+T cells. Additionally, the presence of *Algibacter* was inversely correlated with the expression of key chemokines, such as C-X-C motif ligand (CXCL)9, CXCL10, and the C-C motif chemokine ligand 5, which play a crucial role in T cell trafficking and function (22). A notable correlation was identified between the presence of microorganisms in the tumor and the infiltration of regulatory T cells (77). Analysis of CRC tissues revealed a positive correlation between the abundance of microorganisms in the tumor core and the extent of CD3+T cell infiltration. Furthermore, their findings showed that the tumor cores of patients with elevated tumor-infiltrating lymphocytes (TILs) levels were enriched with nine distinct bacterial species (78). Intratumor microbiota directly interacts with pattern recognition receptors, such as toll-like receptors (TLRs), on both tumor and immune cells, triggering downstream signaling pathways that activate pro-inflammatory mediators (cytokines and chemokines), potentially fueling tumor growth and immune modulation (31, 79, 80). *Fusobacterium nucleatum*, a well-studied bacterium in cancer pathogenesis, can bind to TLR4 on cancer cells and activate the nuclear factor-kappaB (NF-κB) signaling cascade, leading to the production of pro-inflammatory cytokines such as interleukin (IL)-1β, IL-6, and tumor necrosis factor -α (TNF-α), which promote a chronic inflammatory state (81). Intratumor microbiota may also contribute to immune evasion in thyroid cancer by modulating the activity of immune cells, particularly T cells and natural killer (NK) cells. *Fusobacterium nucleatum* has been shown to impair NK cell function via its Fap2 protein, which binds to the T cell immunoglobulin and ITIM domain (TIGIT) receptor on NK cells, inhibiting their cytotoxic activity against tumor cells (82). *Fusobacterium nucleatum* and other bacteria, such as *Methylobacterium*, have been associated with a reduction in TILs, particularly CD8+T cells and dysfunction of T cell in various cancers, including colorectal and breast cancers. Reduced TILs’ presence and impaired T cell function create an immune-suppressive TME, allowing tumor cells to evade immune surveillance and promoting tumor progression (83–85). Bacterial-derived ROS can modulate immune responses by altering the function of immune cells within the TME. ROS may impair the activity of cytotoxic T cells and NK cells, reducing the immune system’s ability to target and eliminate cancer cells (77, 79, 86). Microbial metabolites, such as lipopolysaccharide (LPS), short-chain fatty acids (SCFAs) and other bacterial byproducts, can have profound effects on the immune landscape of the tumor. LPS, a component of the outer membrane of Gram-negative bacteria, is a potent activator of TLR4 signaling (87). By binding to TLR4 on immune cells and tumor cells, LPS may induce the production of pro-inflammatory cytokines and chemokines, thereby driving chronic inflammation within the TME (31, 88). This inflammation not only supports tumor growth but also fosters an immune-suppressive environment, wherein immune cells are less effective at mounting an anti-tumor response. Moreover, LPS-induced NF-κB activation has been shown to upregulate the expression of miR-21, a microRNA that promotes tumor cell proliferation and survival by inhibiting tumor suppressor pathways, such as the RAS signaling pathway (81). SCFAs, such as butyrate, have been implicated in regulating immune cell differentiation and function (89). Exposure to culture supernatants of *Megasphaera massiliensis*, which contain high levels of the SCFAs, enhanced the production of IFN-γ and TNF-α in CD8+T cells. Furthermore, these treated cytotoxic CD8+T cells exhibited superior tumor reactivity and *in vivo* persistence compared to their untreated counterparts when administered as adoptive cell therapy in a murine model of melanoma (90).

Immune checkpoints play a pivotal role in regulating immune evasion and tumor progression. In thyroid cancer, including aggressive subtypes like ATC, the upregulation of these immune checkpoint proteins such as Programmed death-1 (PD-1), programmed death-ligand 1 (PD-L1) has been linked to poorer prognosis and enhanced tumor invasiveness (91, 92). Recent advances highlight that intratumor microbiota directly modulate immune checkpoint pathways within the TME, influencing both tumor immunogenicity and immunotherapy efficacy. Intratumoral *Fusobacterium nucleatum* could enhance anti-PD-1 efficacy in microsatellite stable CRC by suppressing PD-1 overexpression in CD8+ TILs through the butyric acid- histone deacetylase 3/8-TBX21 axis, thereby restoring anti-tumor immunity (93). The elimination of intratumor microbiota could improve the therapeutic effectiveness of α-PD-L1 immunotherapy (94). It is conceivable that intratumor microbiota might influence the response to ICIs in thyroid cancer as well, either by promoting an immune-suppressive TME or by directly modulating immune checkpoint pathways. Further research is needed to explore how bacterial presence and activity within thyroid cancer might impact the efficacy of immunotherapies.

The interactions between intratumor microbiota and the TME are complex, involving the modulation of immune responses, activation of signaling pathways, and the generation of pro-tumorigenic metabolites. The mechanistic roles of intratumor microbiota in thyroid carcinogenesis remain underexplored compared to other malignancies such as CRC or pancreatic cancer. While emerging evidence from non-thyroid cancers implicates microbiota-driven pathways (e.g., Wnt/β-catenin, TLR/NF-κB) in tumor progression and therapy resistance, direct experimental validation in thyroid cancer models is lacking. We explicitly emphasize that these pathways remain speculative in thyroid cancer and warrant rigorous investigation using patient-derived organoids, germ-free animal models, and microbiota-depletion approaches. Future studies should aim to identify the bacterial species and mechanisms contributing to thyroid cancer development and investigate the potential of microbiota-targeted therapies.

## 6 Conclusions and future perspectives

Recent advancements in detection techniques have significantly improved the rapid identification of microbes residing within cancerous tissues. The role of intratumor microbiota in cancer pathogenesis is a rapidly expanding field, with numerous studies highlighting how these microbial communities may influence cancer initiation, progression and treatments. Microorganisms, once established in tumors, may promote tumorigenesis by enhancing mutation rates, modulating oncogenic signaling pathways, and altering the TME. Targeting specific microbial populations or their metabolic products may enhance the efficacy of existing therapies or pave the way for innovative microbiota-based treatments. Although current evidence supports a correlation between intratumor microbiota and various tumor characteristics, these associations should not be interpreted as causative, and mechanistic insights remain limited without direct functional evidence. Most of the existing studies are observational in nature, and the relationship between microbiota and tumor progression or immune modulation remains to be elucidated.

In thyroid cancer, the discovery of intratumor microbiota presents an intriguing new dimension to understanding its pathogenesis and progression. While research in other cancer types has uncovered valuable insights regarding microbial origin, diversity, and functional roles, much remains to be explored in the context of thyroid cancer, particularly with respect to their therapeutic implications. Disrupting specific cancer-promoting or immunosuppressive microbial populations within tumors could potentially interfere with tumorigenic pathways, modulate the TME, and improve treatment outcomes. Targeting the intratumor microbiota has emerged as a promising strategy for improving treatment efficacy. Probiotics may help restore microbial homeostasis, potentially mitigating pro-tumorigenic effects, while antibiotics could selectively eliminate harmful bacterial species. Additionally, microbiome modulation through dietary interventions or fecal microbiota transplantation may offer novel therapeutic avenues. A deeper understanding of these microbial interactions is essential for optimizing personalized treatment strategies. Future research should focus on identifying specific bacterial signatures associated with thyroid cancer and evaluating the clinical benefits of microbiota-targeted therapies. Integrating microbiome-based approaches with existing treatment modalities may pave the way for more effective and tailored therapeutic options. Additionally, utilizing the intratumor microbiota as a biomarker for cancer diagnosis and prognosis holds promise for advancing personalized medicine.

Moreover, the present literature is largely focused on PTC, with relatively few studies addressing other histological subtypes such as FTC, MTC, and ATC. Future research should aim to explore the microbiota landscape in these subtypes to provide a more comprehensive understanding of the thyroid tumor microenvironment.

Future research on the intratumor microbiota in thyroid cancer could significantly advance new diagnostic and therapeutic approaches. However, several challenges remain. It remains uncertain whether particular microbial changes are a cause or effect of tumor development, or if they occur incidentally. Second, the minimal biomass of tumor-associated microbial communities and the potential for contamination during sample collection and processing highlight the necessity for rigorous controls and standardized protocols to ensure study reproducibility and comparability. Third, a critical consideration in interpreting intratumor microbiota studies lies in the inherent limitations and technical biases of current detection methodologies. While 16S rRNA sequencing remains widely used for bacterial profiling, taxonomic biases still exist. Shotgun metagenomics, though offering strain-level resolution, struggles with low microbial biomass in tumors, where host DNA contamination obscures bacterial signals. To address these challenges, future studies should integrate orthogonal methods. Single-cell RNA sequencing (scRNA-seq) offers unprecedented insights into the cellular heterogeneity of the TME and the specific roles of individual immune cells in relation to microbial presence. This technique allows researchers to dissect the complex interactions between immune cells and intratumor microbiota at a single-cell resolution, providing a clearer picture of how these interactions may influence cancer progression and response to therapy. Additionally, spatial transcriptomics has emerged as a powerful tool to study microbial localization within tumor niches. By mapping gene expression data spatially, this method enables researchers to visualize the precise locations of microbial communities and their interactions with surrounding cells. Understanding the spatial dynamics of intratumor microbiota can reveal new therapeutic targets and improve the design of microbiota-based interventions. Incorporating these cutting-edge methodologies into thyroid cancer research will likely drive significant advancements in our understanding of the microbiota-cancer axis, paving the way for innovative treatment strategies. Fourth, further investigation is necessary to clarify the roles of individual microbial species and their interactions with the TME. Fifth, existing studies on intratumor bacteria’s role in thyroid cancer have mainly concentrated on PTC. In contrast, studies investigating the involvement of intratumor bacteria in other thyroid cancer types are relatively scarce. Finally, translating findings from animal models, particularly mice, to human applications poses a challenge, given the significant interspecies differences that can complicate such efforts. In conclusion, despite significant advancements in understanding intratumor microbiota’s role in thyroid cancer, future research must address existing challenges to fully harness microbiota-based diagnostics and therapies in clinical settings.

## References

[1] Siegel RL Giaquinto AN Jemal A. Cancer statistics, 2024. CA Cancer J Clin. (2024) 74:12–49. doi: 10.3322/caac.21820 38230766

[2] Boucai L Zafereo M Cabanillas ME. Thyroid cancer: A review. Jama. (2024) 331:425–35. doi: 10.1001/jama.2023.26348 38319329

[3] Laha D Nilubol N Boufraqech M. New therapies for advanced thyroid cancer. Front Endocrinol (Lausanne). (2020) 11:82. doi: 10.3389/fendo.2020.00082 32528402 PMC7257776

[4] Saenko V Mitsutake N. Radiation-related thyroid cancer. Endocr Rev. (2024) 45:1–29. doi: 10.1210/endrev/bnad022 37450579 PMC10765163

[5] Shobab L Burman KD Wartofsky L. Sex differences in differentiated thyroid cancer. Thyroid. (2022) 32:224–35. doi: 10.1089/thy.2021.0361 34969307

[6] Fagin JA Nikiforov YE. Progress in thyroid cancer genomics: A 40-year journey. Thyroid. (2023) 33:1271–86. doi: 10.1089/thy.2023.0045 PMC10664575 37668657

[7] Manzella L Stella S Pennisi MS Tirrò E Massimino M Romano C. New insights in thyroid cancer and p53 family proteins. Int J Mol Sci. (2017) 18:1325. doi: 10.3390/ijms18061325 28635633 PMC5486146

[8] Lasolle H Schiavo A Tourneur A Gillotay P de Faria da Fonseca B Ceolin L. Dual targeting of MAPK and PI3K pathways unlocks redifferentiation of Braf-mutated thyroid cancer organoids. Oncogene. (2024) 43:155–70. doi: 10.1038/s41388-023-02889-y PMC10786723 37985676

[9] Zhang X Luo B Sun M Gao D Xu S. Research progress of DNA methylation in the diagnosis and treatment of thyroid carcinoma. Int Immunopharmacol. (2025) 152:114426. doi: 10.1016/j.intimp.2025.114426 40058105

[10] Chen C Liu J. Histone acetylation modifications: A potential targets for the diagnosis and treatment of papillary thyroid cancer. Front Oncol. (2022) 12:1053618. doi: 10.3389/fonc.2022.1053618 36523971 PMC9745171

[11] Tiedje V Fagin JA. Therapeutic breakthroughs for metastatic thyroid cancer. Nat Rev Endocrinol. (2020) 16:77–8. doi: 10.1038/s41574-019-0307-2 PMC7470005 31819229

[12] Liu S Gao J Zhu M Liu K Zhang HL. Gut microbiota and dysbiosis in alzheimer’s disease: implications for pathogenesis and treatment. Mol Neurobiol. (2020) 57:5026–43. doi: 10.1007/s12035-020-02073-3 PMC7541367 32829453

[13] Structure, function and diversity of the healthy human microbiome. Nature. (2012) 486:207–14. doi: 10.1038/nature11234 PMC3564958 22699609

[14] Sepich-Poore GD Zitvogel L Straussman R Hasty J Wargo JA Knight R. The microbiome and human cancer. Science. (2021) 371:eabc4552. doi: 10.1126/science.abc4552 33766858 PMC8767999

[15] Herrera-Quintana L Vázquez-Lorente H Lopez-Garzon M Cortés-Martín A Plaza-Diaz J. Cancer and the microbiome of the human body. Nutrients. (2024) 16:2790. doi: 10.3390/nu16162790 39203926 PMC11357655

[16] Li W Deng Y Chu Q Zhang P. Gut microbiome and cancer immunotherapy. Cancer Lett. (2019) 447:41–7. doi: 10.1016/j.canlet.2019.01.015 30684593

[17] Zhou CB Zhou YL Fang JY. Gut microbiota in cancer immune response and immunotherapy. Trends Cancer. (2021) 7:647–60. doi: 10.1016/j.trecan.2021.01.010 33674230

[18] Poore GD Kopylova E Zhu Q Carpenter C Fraraccio S Wandro S. Microbiome analyses of blood and tissues suggest cancer diagnostic approach. Nature. (2020) 579:567–74. doi: 10.1038/s41586-020-2095-1 PMC7500457 32214244

[19] Nejman D Livyatan I Fuks G Gavert N Zwang Y Geller LT. The human tumor microbiome is composed of tumor type-specific intracellular bacteria. Science. (2020) 368:973–80. doi: 10.1126/science.aay9189 PMC7757858 32467386

[20] Yang L Li A Wang Y Zhang Y. Intratumoral microbiota: roles in cancer initiation, development and therapeutic efficacy. Signal Transduct Target Ther. (2023) 8:35. doi: 10.1038/s41392-022-01304-4 36646684 PMC9842669

[21] Che S Yan Z Feng Y Zhao H. Unveiling the intratumoral microbiota within cancer landscapes. iScience. (2024) 27:109893. doi: 10.1016/j.isci.2024.109893 38799560 PMC11126819

[22] Zhu G Su H Johnson CH Khan SA Kluger H Lu L. Intratumour microbiome associated with the infiltration of cytotoxic CD8+ T cells and patient survival in cutaneous melanoma. Eur J Cancer. (2021) 151:25–34. doi: 10.1016/j.ejca.2021.03.053 33962358 PMC8184628

[23] Bi X Wang J Liu C. Intratumoral microbiota: metabolic influences and biomarker potential in gastrointestinal cancer. Biomolecules. (2024) 14:917. doi: 10.3390/biom14080917 39199305 PMC11353126

[24] Xue C Chu Q Zheng Q Yuan X Su Y Bao Z. Current understanding of the intratumoral microbiome in various tumors. Cell Rep Med. (2023) 4:100884. doi: 10.1016/j.xcrm.2022.100884 36652905 PMC9873978

[25] John D Yalamarty R Barakchi A Chen T Chakladar J Li WT. Transcriptomic analysis reveals dysregulation of the mycobiome and archaeome and distinct oncogenic characteristics according to subtype and gender in papillary thyroid carcinoma. Int J Mol Sci. (2023) 24:3148. doi: 10.3390/ijms24043148 36834564 PMC9967748

[26] Fox JG Dewhirst FE Tully JG Paster BJ Yan L Taylor NS. Helicobacter hepaticus sp. nov., a microaerophilic bacterium isolated from livers and intestinal mucosal scrapings from mice. J Clin Microbiol. (1994) 32:1238–45. doi: 10.1128/jcm.32.5.1238-1245.1994 PMC263656 8051250

[27] Galeano Niño JL Wu H LaCourse KD Kempchinsky AG Baryiames A Barber B. Effect of the intratumoral microbiota on spatial and cellular heterogeneity in cancer. Nature. (2022) 611:810–7. doi: 10.1038/s41586-022-05435-0 PMC9684076 36385528

[28] Narunsky-Haziza L Sepich-Poore GD Livyatan I Asraf O Martino C Nejman D. Pan-cancer analyses reveal cancer-type-specific fungal ecologies and bacteriome interactions. Cell. (2022) 185:3789–806.e17. doi: 10.1016/j.cell.2022.09.005 36179670 PMC9567272

[29] Ramírez-Labrada AG Isla D Artal A Arias M Rezusta A Pardo J. The influence of lung microbiota on lung carcinogenesis, immunity, and immunotherapy. Trends Cancer. (2020) 6:86–97. doi: 10.1016/j.trecan.2019.12.007 32061309

[30] Li ZR Li J Cai W Lai JYH McKinnie SMK Zhang WP. Macrocyclic colibactin induces DNA double-strand breaks via copper-mediated oxidative cleavage. Nat Chem. (2019) 11:880–9. doi: 10.1038/s41557-019-0317-7 PMC6761029 31527851

[31] Pushalkar S Hundeyin M Daley D Zambirinis CP Kurz E Mishra A. The pancreatic cancer microbiome promotes oncogenesis by induction of innate and adaptive immune suppression. Cancer Discov. (2018) 8:403–16. doi: 10.1158/2159-8290.CD-17-1134 PMC6225783 29567829

[32] Eisenhofer R Minich JJ Marotz C Cooper A Knight R Weyrich LS. Contamination in low microbial biomass microbiome studies: issues and recommendations. Trends Microbiol. (2019) 27:105–17. doi: 10.1016/j.tim.2018.11.003 30497919

[33] Weiss S Amir A Hyde ER Metcalf JL Song SJ Knight R. Tracking down the sources of experimental contamination in microbiome studies. Genome Biol. (2014) 15:564. doi: 10.1186/s13059-014-0564-2 25608874 PMC4311479

[34] Zheng HH Du CT Yu C Tang XY Huang RL Zhang YZ. The relationship of tumor microbiome and oral bacteria and intestinal dysbiosis in canine mammary tumor. Int J Mol Sci. (2022) 23:10928. doi: 10.3390/ijms231810928 36142841 PMC9503607

[35] Siggins MK Lynskey NN Lamb LE Johnson LA Huse KK Pearson M. Extracellular bacterial lymphatic metastasis drives Streptococcus pyogenes systemic infection. Nat Commun. (2020) 11:4697. doi: 10.1038/s41467-020-18454-0 32943639 PMC7498588

[36] Schorr L Mathies M Elinav E Puschhof J. Intracellular bacteria in cancer-prospects and debates. NPJ Biofilms Microbiomes. (2023) 9:76. doi: 10.1038/s41522-023-00446-9 37813921 PMC10562400

[37] Bertocchi A Carloni S Ravenda PS Bertalot G Spadoni I Lo Cascio A. Gut vascular barrier impairment leads to intestinal bacteria dissemination and colorectal cancer metastasis to liver. Cancer Cell. (2021) 39:708–24.e11. doi: 10.1016/j.ccell.2021.03.004 33798472

[38] Fu A Yao B Dong T Chen Y Yao J Liu Y. Tumor-resident intracellular microbiota promotes metastatic colonization in breast cancer. Cell. (2022) 185:1356–72.e26. doi: 10.1016/j.cell.2022.02.027 35395179

[39] Liu CJ Chen SQ Zhang SY Wang JL Tang XD Yang KX. The comparison of microbial communities in thyroid tissues from thyroid carcinoma patients. J Microbiol. (2021) 59:988–1001. doi: 10.1007/s12275-021-1271-9 34613604

[40] Huang J Mao Y Wang L. The crosstalk of intratumor bacteria and the tumor. Front Cell Infect Microbiol. (2023) 13:1273254. doi: 10.3389/fcimb.2023.1273254 38235490 PMC10791805

[41] Byrd DA Fan W Greathouse KL Wu MC Xie H Wang X. The intratumor microbiome is associated with microsatellite instability. J Natl Cancer Inst. (2023) 115:989–93. doi: 10.1093/jnci/djad083 PMC10407713 37192013

[42] Yang X An H He Y Fu G Jiang Z. Comprehensive analysis of microbiota signature across 32 cancer types. Front Oncol. (2023) 13:1127225. doi: 10.3389/fonc.2023.1127225 36969036 PMC10031003

[43] Guo W Zhang Y Guo S Mei Z Liao H Dong H. Tumor microbiome contributes to an aggressive phenotype in the basal-like subtype of pancreatic cancer. Commun Biol. (2021) 4:1019. doi: 10.1038/s42003-021-02557-5 34465850 PMC8408135

[44] Liu W Zhang X Xu H Li S Lau HC Chen Q. Microbial community heterogeneity within colorectal neoplasia and its correlation with colorectal carcinogenesis. Gastroenterology. (2021) 160:2395–408. doi: 10.1053/j.gastro.2021.02.020 33581124

[45] Tudhope GR Wilson GM. Anaemia in hypothyroidism. Incidence, pathogenesis, and response to treatment. Q J Med. (1960) 29:513–37. 13778548

[46] Cellini M Santaguida MG Virili C Capriello S Brusca N Gargano L. Hashimoto’s thyroiditis and autoimmune gastritis. Front Endocrinol (Lausanne). (2017) 8:92. doi: 10.3389/fendo.2017.00092 28491051 PMC5405068

[47] Dai D Yang Y Yang Y Dang T Xiao J Wang W. Alterations of thyroid microbiota across different thyroid microhabitats in patients with thyroid carcinoma. J Transl Med. (2021) 19:488. doi: 10.1186/s12967-021-03167-9 34847917 PMC8638380

[48] Yuan L Yang P Wei G Hu X Chen S Lu J. Tumor microbiome diversity influences papillary thyroid cancer invasion. Commun Biol. (2022) 5:864. doi: 10.1038/s42003-022-03814-x 36002642 PMC9402670

[49] Gnanasekar A Castaneda G Iyangar A Magesh S Perez D Chakladar J. The intratumor microbiome predicts prognosis across gender and subtypes in papillary thyroid carcinoma. Comput Struct Biotechnol J. (2021) 19:1986–97. doi: 10.1016/j.csbj.2021.03.032 PMC8085784 33995898

[50] Li Z Zhou J Liang H Ye L Lan L Lu F. Differences in alpha diversity of gut microbiota in neurological diseases. Front Neurosci. (2022) 16:879318. doi: 10.3389/fnins.2022.879318 35837118 PMC9274120

[51] Yang Y Gan M Yi K Han S Lin Z Shi Y. Guiding the postoperative radioactive iodine-131 therapy for patients with papillary thyroid carcinoma according to the prognostic risk groups: a SEER-based study. J Cancer Res Clin Oncol. (2023) 149:17147–57. doi: 10.1007/s00432-023-05299-5 PMC11796916 37782329

[52] Shivaprasad KS Siddardha K. Pituitary hyperplasia from primary hypothyroidism. N Engl J Med. (2019) 380:e9. doi: 10.1056/NEJMicm1805378 30786191

[53] Xu B Gu SY Zhou NM Jiang JJ. Association between thyroid stimulating hormone levels and papillary thyroid cancer risk: A meta-analysis. Open Life Sci. (2023) 18:20220671. doi: 10.1515/biol-2022-0671 37588997 PMC10426723

[54] Klapp V Álvarez-Abril B Leuzzi G Kroemer G Ciccia A Galluzzi L. The DNA damage response and inflammation in cancer. Cancer Discov. (2023) 13:1521–45. doi: 10.1158/2159-8290.CD-22-1220 37026695

[55] Gielecińska A Kciuk M Kołat D Kruczkowska W Kontek R. Polymorphisms of DNA repair genes in thyroid cancer. Int J Mol Sci. (2024) 25:5995. doi: 10.3390/ijms25115995 38892180 PMC11172789

[56] Greathouse KL White JR Vargas AJ Bliskovsky VV Beck JA von Muhlinen N. Interaction between the microbiome and TP53 in human lung cancer. Genome Biol. (2018) 19:123. doi: 10.1186/s13059-018-1501-6 30143034 PMC6109311

[57] Backert S Linz B Tegtmeyer N. Helicobacter pylori-induced host cell DNA damage and genetics of gastric cancer development. Curr Top Microbiol Immunol. (2023) 444:185–206. doi: 10.1007/978-3-031-47331-9 38231219

[58] Tronnet S Oswald E. Quantification of colibactin-associated genotoxicity in heLa cells by in cell western (ICW) using γ-H2AX as a marker. Bio Protoc. (2018) 8:e2771. doi: 10.21769/BioProtoc.2771 PMC8203971 34179287

[59] Nougayrède JP Homburg S Taieb F Boury M Brzuszkiewicz E Gottschalk G. Escherichia coli induces DNA double-strand breaks in eukaryotic cells. Science. (2006) 313:848–51. doi: 10.1126/science.1127059 16902142

[60] Pleguezuelos-Manzano C Puschhof J Rosendahl Huber A van Hoeck A Wood HM Nomburg J. Mutational signature in colorectal cancer caused by genotoxic pks(+) E. coli. Nature. (2020) 580:269–73. doi: 10.1038/s41586-020-2080-8 PMC8142898 32106218

[61] La Rosa GRM Gattuso G Pedullà E Rapisarda E Nicolosi D Salmeri M. Association of oral dysbiosis with oral cancer development. Oncol Lett. (2020) 19:3045–58. doi: 10.3892/ol.2020.11441 PMC7079586 32211076

[62] Kryston TB Georgiev AB Pissis P Georgakilas AG. Role of oxidative stress and DNA damage in human carcinogenesis. Mutat Res. (2011) 711:193–201. doi: 10.1016/j.mrfmmm.2010.12.016 21216256

[63] Ozben T. Oxidative stress and apoptosis: impact on cancer therapy. J Pharm Sci. (2007) 96:2181–96. doi: 10.1002/jps.20874 17593552

[64] Chung L Thiele Orberg E Geis AL Chan JL Fu K DeStefano Shields CE. Bacteroides fragilis Toxin Coordinates a Pro-carcinogenic Inflammatory Cascade via Targeting of Colonic Epithelial Cells. Cell Host Microbe. (2018) 23:203–14.e5. doi: 10.1016/j.chom.2018.01.007 29398651 PMC5954996

[65] Goodwin AC Destefano Shields CE Wu S Huso DL Wu X Murray-Stewart TR. Polyamine catabolism contributes to enterotoxigenic Bacteroides fragilis-induced colon tumorigenesis. Proc Natl Acad Sci U S A. (2011) 108:15354–9. doi: 10.1073/pnas.1010203108 PMC3174648 21876161

[66] van der Veen S Tang CM. The BER necessities: the repair of DNA damage in human-adapted bacterial pathogens. Nat Rev Microbiol. (2015) 13:83–94. doi: 10.1038/nrmicro3391 25578955

[67] Motegi A Masutani M Yoshioka KI Bessho T. Aberrations in DNA repair pathways in cancer and therapeutic significances. Semin Cancer Biol. (2019) 58:29–46. doi: 10.1016/j.semcancer.2019.02.005 30922960

[68] Prete A Borges de Souza P Censi S Muzza M Nucci N Sponziello M. Update on fundamental mechanisms of thyroid cancer. Front Endocrinol (Lausanne). (2020) 11:102. doi: 10.3389/fendo.2020.00102 32231639 PMC7082927

[69] Rubinstein MR Wang X Liu W Hao Y Cai G Han YW. Fusobacterium nucleatum promotes colorectal carcinogenesis by modulating E-cadherin/β-catenin signaling via its FadA adhesin. Cell Host Microbe. (2013) 14:195–206. doi: 10.1016/j.chom.2013.07.012 23954158 PMC3770529

[70] Rubinstein MR Baik JE Lagana SM Han RP Raab WJ Sahoo D. Fusobacterium nucleatum promotes colorectal cancer by inducing Wnt/β-catenin modulator Annexin A1. EMBO Rep. (2019) 20:e47638. doi: 10.15252/embr.201847638 30833345 PMC6446206

[71] Lu R Wu S Zhang YG Xia Y Liu X Zheng Y. Enteric bacterial protein AvrA promotes colonic tumorigenesis and activates colonic beta-catenin signaling pathway. Oncogenesis. (2014) 3:e105. doi: 10.1038/onc.2012.545 24911876 PMC4150214

[72] Song X Xin N Wang W Zhao C. Wnt/β-catenin, an oncogenic pathway targeted by H. pylori in gastric carcinogenesis. Oncotarget. (2015) 6:35579–88. doi: 10.18632/oncotarget.5758 PMC4742126 26417932

[73] Parida S Wu S Siddharth S Wang G Muniraj N Nagalingam A. A procarcinogenic colon microbe promotes breast tumorigenesis and metastatic progression and concomitantly activates notch and β-catenin axes. Cancer Discov. (2021) 11:1138–57. doi: 10.1158/2159-8290.CD-20-0537 33408241

[74] Shin E Koo JS. Cell component and function of tumor microenvironment in thyroid cancer. Int J Mol Sci. (2022) 23:12578. doi: 10.3390/ijms232012578 36293435 PMC9604510

[75] Song M Liu Q Sun W Zhang H. Crosstalk between thyroid carcinoma and tumor-correlated immune cells in the tumor microenvironment. Cancers (Basel). (2023) 15:2863. doi: 10.3390/cancers15102863 37345200 PMC10216712

[76] Ferrari SM Fallahi P Galdiero MR Ruffilli I Elia G Ragusa F. Immune and inflammatory cells in thyroid cancer microenvironment. Int J Mol Sci. (2019) 20:4413. doi: 10.3390/ijms20184413 31500315 PMC6769504

[77] Ma J Gnanasekar A Lee A Li WT Haas M Wang-Rodriguez J. Influence of intratumor microbiome on clinical outcome and immune processes in prostate cancer. Cancers (Basel). (2020) 12:2524. doi: 10.3390/cancers12092524 32899474 PMC7564876

[78] Luu K Ye JY Lagishetty V Liang F Hauer M Sedighian F. Fecal and tissue microbiota are associated with tumor T-cell infiltration and mesenteric lymph node involvement in colorectal cancer. Nutrients. (2023) 15:316. doi: 10.3390/nu15020316 36678187 PMC9861998

[79] Garrett WS. Cancer and the microbiota. Science. (2015) 348:80–6. doi: 10.1126/science.aaa4972 PMC5535753 25838377

[80] Villemin C Six A Neville BA Lawley TD Robinson MJ Bakdash G. The heightened importance of the microbiome in cancer immunotherapy. Trends Immunol. (2023) 44:44–59. doi: 10.1016/j.it.2022.11.002 36464584

[81] Yang Y Weng W Peng J Hong L Yang L Toiyama Y. Fusobacterium nucleatum increases proliferation of colorectal cancer cells and tumor development in mice by activating toll-like receptor 4 signaling to nuclear factor-κB, and up-regulating expression of microRNA-21. Gastroenterology. (2017) 152:851–66.e24. doi: 10.1053/j.gastro.2016.11.018 27876571 PMC5555435

[82] Gur C Ibrahim Y Isaacson B Yamin R Abed J Gamliel M. Binding of the Fap2 protein of Fusobacterium nucleatum to human inhibitory receptor TIGIT protects tumors from immune cell attack. Immunity. (2015) 42:344–55. doi: 10.1016/j.immuni.2015.01.010 PMC4361732 25680274

[83] Parhi L Alon-Maimon T Sol A Nejman D Shhadeh A Fainsod-Levi T. Breast cancer colonization by Fusobacterium nucleatum accelerates tumor growth and metastatic progression. Nat Commun. (2020) 11:3259. doi: 10.1038/s41467-020-16967-2 32591509 PMC7320135

[84] Peng R Liu S You W Huang Y Hu C Gao Y. Gastric microbiome alterations are associated with decreased CD8+ Tissue-resident memory T cells in the tumor microenvironment of gastric cancer. Cancer Immunol Res. (2022) 10:1224–40. doi: 10.1158/2326-6066.CIR-22-0107 35881964

[85] Qiao H Tan XR Li H Li JY Chen XZ Li YQ. Association of intratumoral microbiota with prognosis in patients with nasopharyngeal carcinoma from 2 hospitals in China. JAMA Oncol. (2022) 8:1301–9. doi: 10.1001/jamaoncol.2022.2810 PMC9284409 35834269

[86] Shiao SL Kershaw KM Limon JJ You S Yoon J Ko EY. Commensal bacteria and fungi differentially regulate tumor responses to radiation therapy. Cancer Cell. (2021) 39:1202–13.e6. doi: 10.1016/j.ccell.2021.07.002 34329585 PMC8830498

[87] Bai Y Min R Chen P Mei S Deng F Zheng Z. Disulfiram blocks inflammatory TLR4 signaling by targeting MD-2. Proc Natl Acad Sci U S A. (2023) 120:e2306399120. doi: 10.1073/pnas.2306399120 37487070 PMC10401014

[88] Yang Y Jobin C. Microbial imbalance and intestinal pathologies: connections and contributions. Dis Model Mech. (2014) 7:1131–42. doi: 10.1242/dmm.016428 PMC4174524 25256712

[89] Rossi T Vergara D Fanini F Maffia M Bravaccini S Pirini F. Microbiota-derived metabolites in tumor progression and metastasis. Int J Mol Sci. (2020) 21:5786. doi: 10.3390/ijms21165786 32806665 PMC7460823

[90] Luu M Riester Z Baldrich A Reichardt N Yuille S Busetti A. Microbial short-chain fatty acids modulate CD8(+) T cell responses and improve adoptive immunotherapy for cancer. Nat Commun. (2021) 12:4077. doi: 10.1038/s41467-021-24331-1 34210970 PMC8249424

[91] Capdevila J Wirth LJ Ernst T Ponce Aix S Lin CC Ramlau R. PD-1 blockade in anaplastic thyroid carcinoma. J Clin Oncol. (2020) 38:2620–7. doi: 10.1200/JCO.19.02727 PMC7476256 32364844

[92] Sun L Niu T Zhang Y. Association between thyroid cancer and CTLA-4 gene polymorphisms. Cell Mol Biol (Noisy-le-grand). (2023) 69:31–6. doi: 10.14715/cmb/2023.69.4.5 37329551

[93] Wang X Fang Y Liang W Wong CC Qin H Gao Y. Fusobacterium nucleatum facilitates anti-PD-1 therapy in microsatellite stable colorectal cancer. Cancer Cell. (2024) 42:1729–46.e8. doi: 10.1016/j.ccell.2024.08.019 39303724

[94] Han ZY Fu ZJ Wang YZ Zhang C Chen QW An JX. Probiotics functionalized with a gallium-polyphenol network modulate the intratumor microbiota and promote anti-tumor immune responses in pancreatic cancer. Nat Commun. (2024) 15:7096. doi: 10.1038/s41467-024-51534-z 39154092 PMC11330462

